# Supplementary material for: Transcriptomic Insights into Mechanisms of Early Seed Maturation in the Garden Pea (Pisum sativum L.)
Source: Cells. 2020 Mar 23;9(3):779. doi: 10.3390/cells9030779 (PMC7140803; doi:10.3390/cells9030779)

Sprint−2,10(4)

Global Summary

%DE = 0.26

genes with fdr < 0.2 = 7093 (3539 + / 3554 −)

genes with fdr < 0.1 = 6658 (3330 + / 3328 −)

genes with fdr < 0.05 = 6265 (3165 + / 3100 −)

genes with fdr < 0.01 = 5739 (2900 + / 2839 −)

genes in genesets = 0

<FC> = 0

<t−score> = 0

<p−value> = 0

<fdr> = 0.74

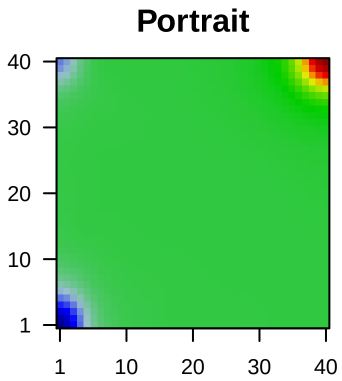

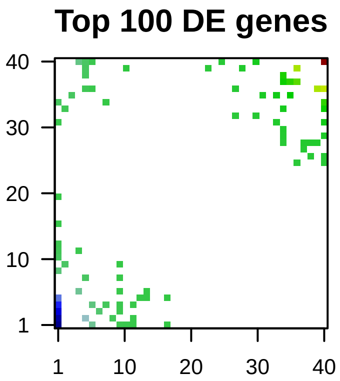

Global Genelist

Rank	ID	log(FC)	p−value / fdr	Metagene	Description
1	TRINITY_DN3	0.06	[illegible]		
2	TRINITY_DN2	−0.06	[illegible]		
3	TRINITY_DN1	−0.11	[illegible]		
4	TRINITY_DN4	0.15	[illegible]		
5	TRINITY_DN4	−0.15	[illegible]		
6	TRINITY_DN3	0.07	[illegible]		
7	TRINITY_DN3	0.07	[illegible]		
8	TRINITY_DN3	0.1	[illegible]		
9	TRINITY_DN3	−0.04	[illegible]		
10	TRINITY_DN3	−0.05	[illegible]		
11	TRINITY_DN3	−0.06	[illegible]		
12	TRINITY_DN3	−0.07	[illegible]		
13	TRINITY_DN1	−0.2	[illegible]		
14	TRINITY_DN1	−0.05	[illegible]		
15	TRINITY_DN1	−0.04	[illegible]		
16	TRINITY_DN1	0.25	[illegible]		
17	TRINITY_DN1	0.13	[illegible]		
18	TRINITY_DN1	0.07	[illegible]		
19	TRINITY_DN1	0.11	[illegible]		
20	TRINITY_DN1	0.78	[illegible]		

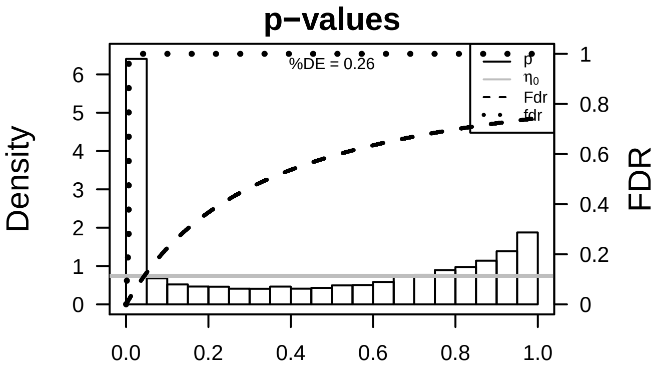

Supplement: Supplementary file 1 [file cells-09-00779-s001.zip › Supplementary materials/FileS2.zip!/FileS1/Summary Sheets - Samples/Sprint.2.10.4..pdf]
